# 60 years of OR in Slovenia: development from a first conference to a vibrant community

**DOI:** 10.1007/s10100-023-00859-z

**Published:** 2023-06-09

**Authors:** Janez Povh, Lidija Zadnik Stirn, Janez Žerovnik

**Affiliations:** 1 Rudolfovo Science and Technology Center Novo Mesto, Novo Mesto, Slovenia; 2 Biotechnical Faculty, University of Ljubljana, Jamnikarjeva 101, 1000 Ljubljana, Slovenia; 3 Faculty of Mechanical Engineering, University of Ljubljana, Aškerčeva 6, 1000 Ljubljana, Slovenia

**Keywords:** History of OR, OR research, SSI-SSOR, SOR symposia

## Abstract

The paper provides a short history of the operations research (OR) in Slovenia. Some major events and achievements are mentioned and briefly discussed. The period starts in the year 1964, with the first symposium on OR in Slovenia. In the following decades, there were several important milestones: (1) the start of master’s and Ph.D. studies in OR in 1974, (2) the establishment of SSI-SSOR in 1992 (SSI-SSOR stands for the Slovenian Section for Operational Research within the Slovenian Society of Informatics), and (3) the start of a series of symposia in operations research in 1993. All these activities resulted in an extensive list of publications, projects, and monographs and international connections, proving that OR is still a vibrant field, which facilitates knowledge transfer from pure research to business applications.

## Beginnings of OR in Slovenia

Although some experts in Slovenia were familiar with the OR before, we may say that the first OR related formal event in Slovenia was the symposium entitled "Mechanografics (Data processing) and OR" with 30 papers, organized in Ljubljana in 1964, by the Slovenian Association of Economists and the Chamber of Commerce of Slovenia. The symposium consisted of two parts, the first part was dealing with the problems of data collection and processing, while the second with OR. In the first part, representatives of various companies and institutions reported on the increase in the volume of data, which requires a more rational way of data acquiring and processing. The presenters reported on experiences and problems in data collection and processing. Some of them also provided practical experiences and theoretical concepts. This duality emphasized the solid connection between theory and practice in the years that followed. In the second part, OR was presented as a new science for decision support in many areas. Several practical examples were given from the fields of industrial production, location, transport, agriculture, investments, and healthcare, in which linear programming and a two-phase production process supported by linear programming dominated as methods. The papers were printed in the proceedings (Mehanografija 1964). This symposium marks the beginning of systematic research, teaching, and consulting work in the field of OR in Slovenia.

In 1967, the Association of Economists of Yugoslavia and the Association of Economists of Slovenia organized at Bled, Slovenia, a conference "Consultation on the use of OR methods in organizations/institutions in Yugoslavia" with 33 papers. A collection of papers was published in proceedings (Posvetovanje 1967). The problems presented at this conference come from production, banking, transport, agriculture, food industry and tourism. Among the methods linear programming was prevailing, while also queuing, networks, branch-and-bound, and dynamic programming methods, and some extensions of simplex method were handled.

The year 1974 marks the beginning of the traditional Yugoslav symposia from OR, known as SYM-OP-IS. These symposia were organized at the Yugoslav level in the organization of the Faculty of Organizational Sciences, the Institute of Industrial Economics, and the Mihajlo Pupin Institute (all from Belgrade). Symposia were held every year in Herceg Novi. Between 150 and 200 participants from all over Yugoslavia took part in them, some from abroad as well. Contributions to SYM-OP-IS symposia were published in proceedings, which were regularly issued as part of each symposium. Researchers and practitioners from Slovenia have regularly participated at these conferences.

In 1974, at the Faculty of Economics of the University of Ljubljana started a master's degree in Operational Research, which some years later grew into a doctoral degree in Information and Management Sciences. This was probably one of the most important milestones in OR development in Slovenia. The students of this study were graduates of economics, mathematics, physics, mechanical engineering, electrical engineering, construction, law, sociology, and others who, after completing their studies, worked or they still work in the field of operational research at universities, institutes and research departments of companies. At the same time, the first book, Outline to operations research (Rupnik 1974), in Slovene language, was published. Unfortunately, these master and PhD study programs have been less and less attractive since 2010. There are also courses in selected OR topics at several faculties in Slovenia, which assure that OR methodology is in active use in engineering, social and natural sciences.

## Foundation of SSI-SSOR and its inclusion into international environment

In 1991, Slovenia and Croatia formally declared independence. In the same year, the Croatian Association of OR (HDOI) was founded and the colleagues from Croatia organized the 1st international OR conference (KOI’91). Many experts from the field of operational research in Slovenia took part at this conference. Following the example of our colleagues from Croatia, at the end of 1992, Slovenia's operational research experts founded their own association, the Slovenian Section for Operational Research (SSOR) under the umbrella of the Slovenian Society of Informatics (SSI), i.e., SSI-SSOR, which has nowadays 107 members. SSI-SSOR is a forum for scientists and practitioners from all areas of OR and allied fields, across the disciplines and programs in resource management, networks, tools, such as linear and nonlinear programming, discrete and combinatorial optimization, stochastic decision-making, multi-criteria optimization, strategic games, inventory theory, graph theory, dynamic optimization, systems management, theory controls and others, information, and education. At its first meeting the founders of SSI-SSOR clearly outlined the goal of the group which is to pursue, support, and facilitate research, development, application, and education in the operations research area where mathematics, economics, computer science, statistics, environmental economics, and system theory, as well as some other disciplines, come together. Pursuing the interdisciplinarity and applied OR science remains the core activity of SSI-SSOR. These include increase the visibility and influence of OR, closer collaboration with educational institutions, industry, government, and international entities, as summarized in Zadnik Stirn and Drobne (2022).

Since the foundation of SSI-SSOR, its members have been active in several fields (Zadnik Stirn 2010). Among the most important activities is the organization of international symposia on operations research in Slovenia, known as SOR’93, SOR’94, …., SOR’21, and the jubilee one, the SOR’23 taking place in September 2023 and devoted to the 30th anniversary of SSI-SSOR. At first, these symposia were organized annually, and later in the year 1997 after an agreement was reached with the Croatian OR Society, they are organized biannually, each year in one country, in Slovenia and Croatia. The members of SSI-SSOR are also co-organizers of annual meetings in informatics which take place in Slovenia, and of the biannually organized symposia on OR in Croatia. They actively participate also in the INFORMS, IFORS, EURO and other OR conferences.

SSI-SSOR is the 47th member of IFORS, admitted to IFORS in 2007 while in the year 2008 it became the 30th member of EURO. It was welcomed to EURO in Sandton during the IFORS conference (Ittmann 2008).

## Review of SSI-SSOR publications

In 1993, SSI-SSOR, with the support of the Ministry of Science and Technology of the Republic of Slovenia and the Faculty of Economics, University of Ljubljana, organized the 1st Symposium on OR, SOR'93, on national level. The Report of SSI-SSOR on SOR'93 is available at https://www.drustvo-informatika.si/sekcije-drustva/SOR/porocilaSOR. In the years 1993–2021, SSI-SSOR organized a total of 16 international symposia in the field of OR. Since 1994, these symposia have been held with international participation, which has been increasing year by year. The symposia had great support from the members of Slovenian universities, the ministries of the Republic of Slovenia, foreign institutions, associations, and societies (Croatian, German, Austrian, Hungarian, Polish OR societies, and others), EURO, IFORS, and many other entities and individuals.

The symposia SOR are the premiere scientific event around OR in Slovenia that are providing an international forum for scientific exchange at the frontiers of OR, mathematics, statistics, economics, engineering, education, environment, computer science, and other fields. Since 1995, SDI-SOR, in agreement with HDOI, has organized a symposium every other year, which means that SDI-SOR and HDOI alternate annually in organizing OR international symposia, one year in Slovenia, the other year in Croatia. Thus, SDI-SOR organized: SOR’93, SOR'94, SOR'95, SOR'97, SOR'99, SOR'01, SOR'03, SOR'05, SOR'07, SOR'09, SOR'11, SOR' 13, SOR'15, SOR'17, SOR'19, SOR'21. The papers presented at these symposia were reviewed and published in Proceedings (2021), (2019), (2017), (2015), (2013), (2011), (2009), (2007), (2005), (2003), (2001), (1999), (1997), (1995), (1994), (1993). Proceedings SOR'05 and later are also available in electronic form on the website http://fgg-web.fgg.uni-lj.si/~/sdrobne/sor/ which can also be accessed from the symposia website https://sor.fov.um.si/publications/. Detailed symposia reports are available at https://www.drustvo-informatika.si/sekcije-drustva. In all 16 Proceedings which are indexed in Current Mathematical Publications, Mathematical Review, Zentralblatt fuer Mathematik/Mathematics Abstracts, MATH on STN Internationaland CompactMath, INSPEC and others, in total 1014 reviewed papers presented at SOR symposia are issued. Maximal number of papers were presented at SOR’21, i.e., 118, while minimal at SOR’95, only 21. The average of presented and in Proceedings published papers is 63,375 papers per symposium. There were all together 100 keynote papers presented by authors coming from 26 countries. The papers at the symposia were divided into sessions. At all symposia 157 sessions were performed, maximal 19 (11 special session and 8 contributed) at SOR’21, average were 9.81 sessions per symposia. There presented and published papers were written by 1768 authors. Maximal number of authors are tracked at SOR’21 (240), minimal at SOR’95 (28), average number of authors is 110.5 per symposium. The authors come from all over the world, of course the most from Slovenia and geographically close countries (Croatia, Hungary, Austria, Italy, Czech Republic, Slovak Republic, Germany, ….). Maximal number of countries is exhibited at SOR’17 (25), minimal at SOR’93 (only 1 as this was a national symposium), the average number of countries per symposium is 12.56.

Most of the members of SSI-SSOR are attached to the universities or institutes. Thus, the members are mostly researchers in operations research and related fields. Some of them, together with their colleagues from abroad, mostly from Croatia, have compiled their work in the form of monographs. Since 1998, SSI-SSOR has published 5 monographs: Rupnik (1998), Rupnik et al. (2000), Zadnik Stirn et al. (2005), Rupnik and Sundać (2005) and Rupnik (2013).

As we can see from references, some monographs or their parts are in Slovene language. Here we would like to add that for professional Slovene language, i.e. Slovene terminology in operations research, the publications in Slovene are indispensable and of great importance. The members of SSI-SSOR take part also by editing and reviewing of the internet interactive IT terminology dictionary named *Islovar* (http://islovar.org/islovar) which is recognized also as a reference dictionary for public users.

In 1994, the SSI-SSOR joined the Austrian, Czech, Croatian, Hungarian and Slovak Societies for Operations Research in the initiative to start a high-quality international journal. Under the leadership of Austrian colleagues, the journal Central European Journal for Operations Research and Economic was initiated and it was published until the end of 1998. In the year 1999 the journal began to be published by Physica, and later by Springer Verlag under the current name Central European journal of Operations Research (CJOR). The journal was first indexed in Web of Science in 2007. Currently, it is ranked on the boarder of the first and second half of journals according to the impact factor in the Web of Science category Operations Research and Management Science (Jablonsky et al. 2022). Since 1997, the members of SSI-SSOR are co-editors of CJOR. In 2011 the first special issue with guest editors coming from SSI-SSOR was issued. In years 2013, 2015, 2017, 2019, and 2021, SSI-SSOR members edited further special issues which were devoted to professional international symposia organized by SSI-SSOR. The bibliometric analysis of papers published in these six SSI-SSOR special issues of CJOR is presented in Kastrin et al. (2021). The authors also analyze and visualize the yearly dynamics of the number of published papers, highlight the most prolific authors and countries, present the papers with the highest impact in term of the number of citations, and examined the keywords co-occurrence network which is decomposed into eight clusters. The current issue of CJOR is again SSI-SSOR special issue and includes 16 papers which are in more detail presented in the next section.

In 2012, SSI-SSOR was invited by the editor of Business Systems Research (BSR) to edit a special issue. *BSR* is a scientific journal focusing on research findings in economics and business systems. Additionally, BSR considers research that combines business and economics with other fields of scientific enquiry, e.g. information systems, mathematics and social sciences. BSR examines a wide variety of business decisions, processes and activities within the actual business setting, as well as within the systems approach framework. Journal is indexed in Scopus, Web of Science (ESCI-WoS), and is indexed at Portal of Croatian Scientific and Professional Journals. Currently, it is indexed as Q3 for Economics, Econometrics and Finance in Scopus. SSI-SSOR edited since 2012 six special issues: in 2012, 2014, 2016, 2018, 2020 and 2022. The special issues of BSR, i.e., SI of the BSR focused on recent advances in Operations Research and Management Science (OR/MS), with a particular emphasis on linking OR/MS with other areas of quantitative and qualitative methods in the context of a multidisciplinary framework (Drobne et al. 2022).

Members of SSI-SSOR are also involved in publication of the SSI journal Uporabna informatika (in Slovene; translation Applied Informatics) https://uporabna-informatika.si/ui which is a Slovenian professional journal in informatics/OR. Its mission is to inform the professional public and users with up-to-date achievements in informatics/OR in Slovenia and worldwide. A special merit of the journal is its information of Slovenian research projects, and European documents which are the basis of trends in informatics/OR and inevitably influence our environment.

## Recent developments: papers in this issue

The diversity of applications of OR is reflected in the selection of papers published in this volume. The special issue call for papers has been synchronized with the organization of the 16th International Symposium on Operations Research in Slovenia, SOR’21. The conference was planned to take place at Bled, but had to be completed online due to limitations caused by the COVID-19 pandemic. However, the call for papers was not restricted to conference participants and all the manuscripts had been subject to the standard review procedure of the journal.

Theoretical foundations of various models have always been an important part of OR, and often in the focus of temporary research. Linear programming with the simplex algorithm, is the first method that marked OR as a new academic discipline. Linear programming may still be seen as the leading methodology of OR. Several papers in this issue deal with various extensions of linear programming. Optimization problems are often subject to various kinds of inexactness or inaccuracy of input data. This leads to studies of varieties of classical problems where uncertainty is modelled with different means. Another usual generalization of the classical problems is the multi-objective programming, which is considered in several papers in this volume.

The development of models and methods for solving the optimization problems to support the decision-making process is continuous. In this volume, several papers study advanced methods for optimization. Nagy and Varga investigate a new primal–dual long-step interior point algorithm for linear optimization (E.-Nagy and Varga 2023). One way of modeling uncertainty is to have input entries in the form of interval data. Multi-objective linear programming problems, in which two kinds of input entries have the form of interval data are considered in Hladik (2023). This article tackles two types of generalization that led to various definitions of efficient solutions. Further generalizations presented in the volume is interactive programming. New possible applications of interactive programming, based on aspiration levels, are provided by Gaspars Wieloch (2023). Linear fractional programming, more precisely, a new iterative method for solving it, is studied in Perić et al. (2023). Interval programming is used to model a transportation problem by Garajova and Rada because the exact values of supply, demand, and the exact transportation costs are not always available for real-world problems (Garajova and Rada 2023). The multistage bipolar method proposed by Trzaskalik (2023) simplifies the procedure of finding the final solution by a multistage decision processes and allows to use single criterion dynamic programming to solve the problem. Rainbow domination is a recently very popular variation of the graph domination problem. A method that provides exact rainbow domination numbers for some graphs, including closed expressions for some infinite families of graphs, is reported by Gabrovšek et al. (2023).

Because of its emphasis on practical applications, it is hard to decide which classification of contributions to OR is more justified. Sometimes, the application seems to be more important or, at least, more appealing, than theoretical considerations. In the years when we are facing a crisis of energy supply, it is of vital importance to predict, for example, electricity consumption, thus hopefully allowing higher quality of service and reliable delivery (Čegovnik et al. 2023). In some sense, the supply of goods is closely related to waste management. With the growing significance of environmental awareness, the role of renewable materials and their reuse and recycling possibilities have become increasingly important. Two models of waste wood processing are considered in Osz et al. (2023). Taxation is one of the most powerful instruments of fiscal policy, affecting economic growth and investments, as well as competitiveness of companies. Optimal tax policy in a specific model is modelled as a Stackelberg game in Lukač (2023a). A twin paper studies optimal taxation of a perfectly competitive firm with Cobb–Douglas production function as a bilevel programming problem (Lukač 2023b). Theoretical considerations start and end with definitions of suitable models, motivated by real needs. Population ageing together with greater prevalence of multimorbidity adds to the need for complex healthcare services. A multi-criteria decision model for assessing health and self-care ability is proposed by Milavec Kapun et al. (2023). The structure of the Hungarian insurance market operated as a monopoly market until 1986 and has seen major changes since then. It has a strong oligopolistic character, and an interesting question raises how close the market is to a state of perfect competition (Varga and Madari 2023). Data mining is the process of knowledge extraction from data with the algorithms that identify hidden relationships and patterns, which are usually not noticeable at first glance. Data mining has become omnipresent in various domains in the recent decade, but its usage in small and medium enterprises (SMEs) is still underrepresented. The authors (Pejič Bach et al. 2023) investigate the determinants of data mining usage in SMEs using the TOE Framework (Technology-Organisation-Environment). Duality in the analysis of monopsony in the labor market is discussed in Krpan (2023). Duality as a way of thinking enables an elegant derivation of a version of Hotelling´s lemma in the monopsony case. The pressure on the speed of information processing ranks business intelligence technologies among the fastest growing decision support tools. The article (Kasparova 2023) applies the unified theory of acceptance and use of technology to verify the factors determining the implementation of business intelligence tools in business processes, especially in decision-making.

## Conclusions

In this paper, we summarize the decades-long development of OR in Slovenia, which started in 1964 with the first OR symposium in Slovenia. An important milestone for the development of OR was the OR master’s and Ph.D. programs at University of Ljubljana, which generated generations of OR specialists who are still important nodes in the OR community. Another important milestone was the founding of the Slovenian OR society in 1993 and the start of a series of operations research symposia SOR that will celebrate their 30th anniversary in 2023. These events, together with joining international OR organizations, are the cornerstones of international relevance of Slovenian OR. The future of Slovenian OR will strongly depend on the ability to motivate new generations to study Operations Research and to conduct research in OR or in other scientific domains using OR methodology.

For this, the continuation of the SOR series is very important, as well as a strategic approach to attracting new talents in the field of OR.
